# Supplementary figures and images for: Integrating single-cell and bulk sequencing data to identify glycosylation-based genes in non-alcoholic fatty liver disease-associated hepatocellular carcinoma
Source: PeerJ. 2024 Mar 18;12:e17002. doi: 10.7717/peerj.17002 (PMC10956522; doi:10.7717/peerj.17002)

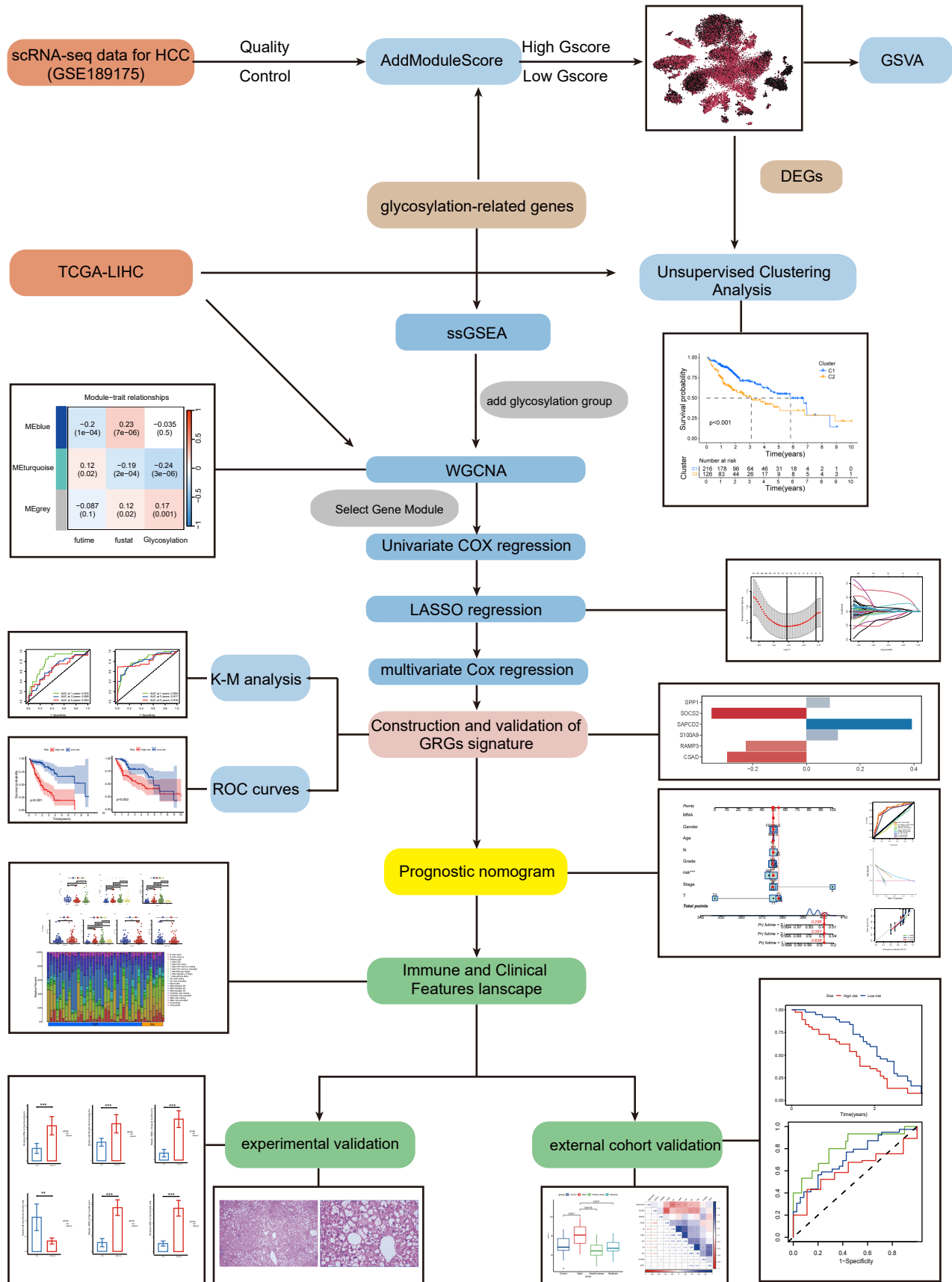

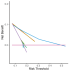

Supplement: Supplemental Information 2 — Our aim was to identify glycosylation hub genes with prognostic significance in NAFLD-associated HCC by a combinatorial algorithm, to build a prognostic prediction model, and to validate its performance and biological significance. Through several steps: 1) scoring all cells through 636 GRGs (Table S1) using the AddModuleScore function Seurat R package to identify differential genes in the scRNA-seq dataset; 2) based on these differentially expressed genes, using an unsupervised clustering algorithm to categorize patients with hepatocellular carcinoma in the TCGA database into two groups, in order to to verify that glycosylation levels can affect patients’ prognosis; 3) Using GRGs as a reference, the ssGSEA algorithm was used to calculate the glycosylation levels of each patient in TCGA-LIHC, and then WGCNA was used to screen out the modules that were highly correlated with the prognosis and glycosylation levels. 4) Then use univariate cox-lasso-Multivariate cox regression to screen the features among modules and build models that can predict the prognosis of HCC patients. 5) Divide the TCGA into a training set and an internal validation set to validate the performance of the models. 6) Construct a column-line graph. 7) Explore the clinical features and immune infiltration of different risk models. 8) In the external validation set (1 hepatocellular carcinoma and 2 NAFLD) sets, animal models and human liver tissues to validate the biological significance of the model features. [file peerj-12-17002-s002.pdf]

(A)

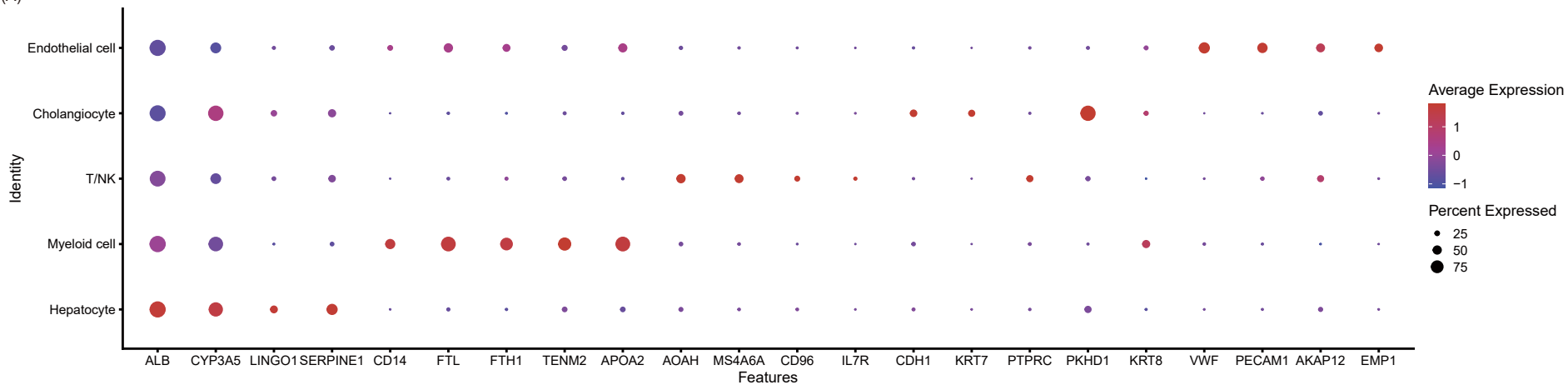

(B)

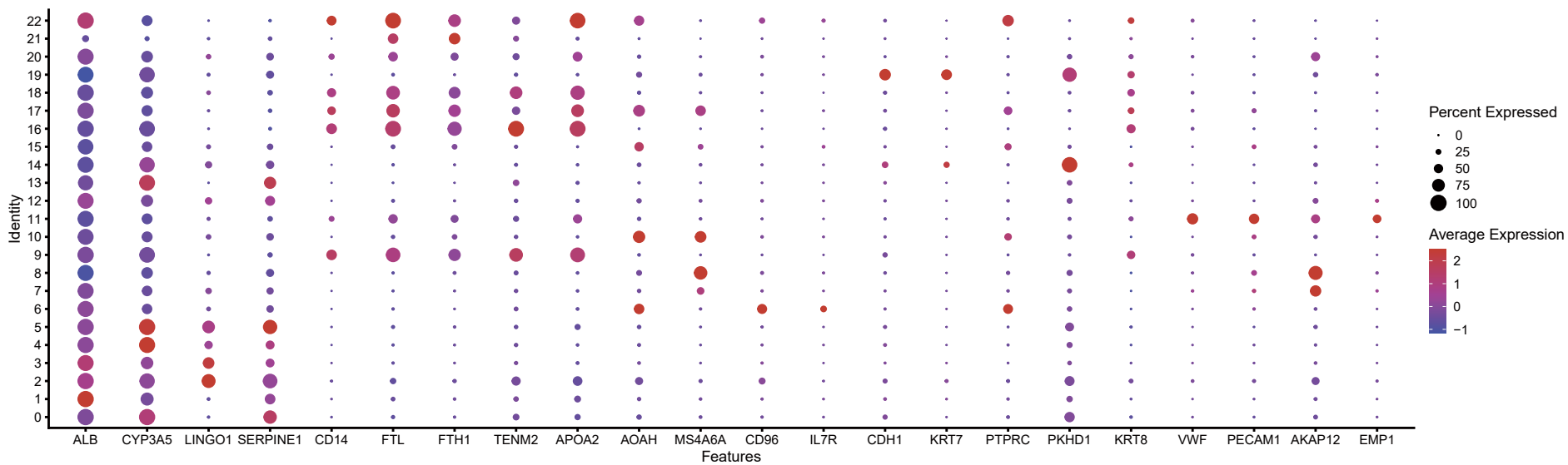

Supplement: Supplemental Information 3 — Dot plot showing marker genes for the five cell types defined in the article. The size of the dots indicates the average proportion of cells expressing the target gene, while the color represents the average expression. (B) Dot plot indicating marker genes for 23 distinct clusters. The meaning of the size and color of the dots in the figure is the same as above. [file peerj-12-17002-s003.pdf]

(A)

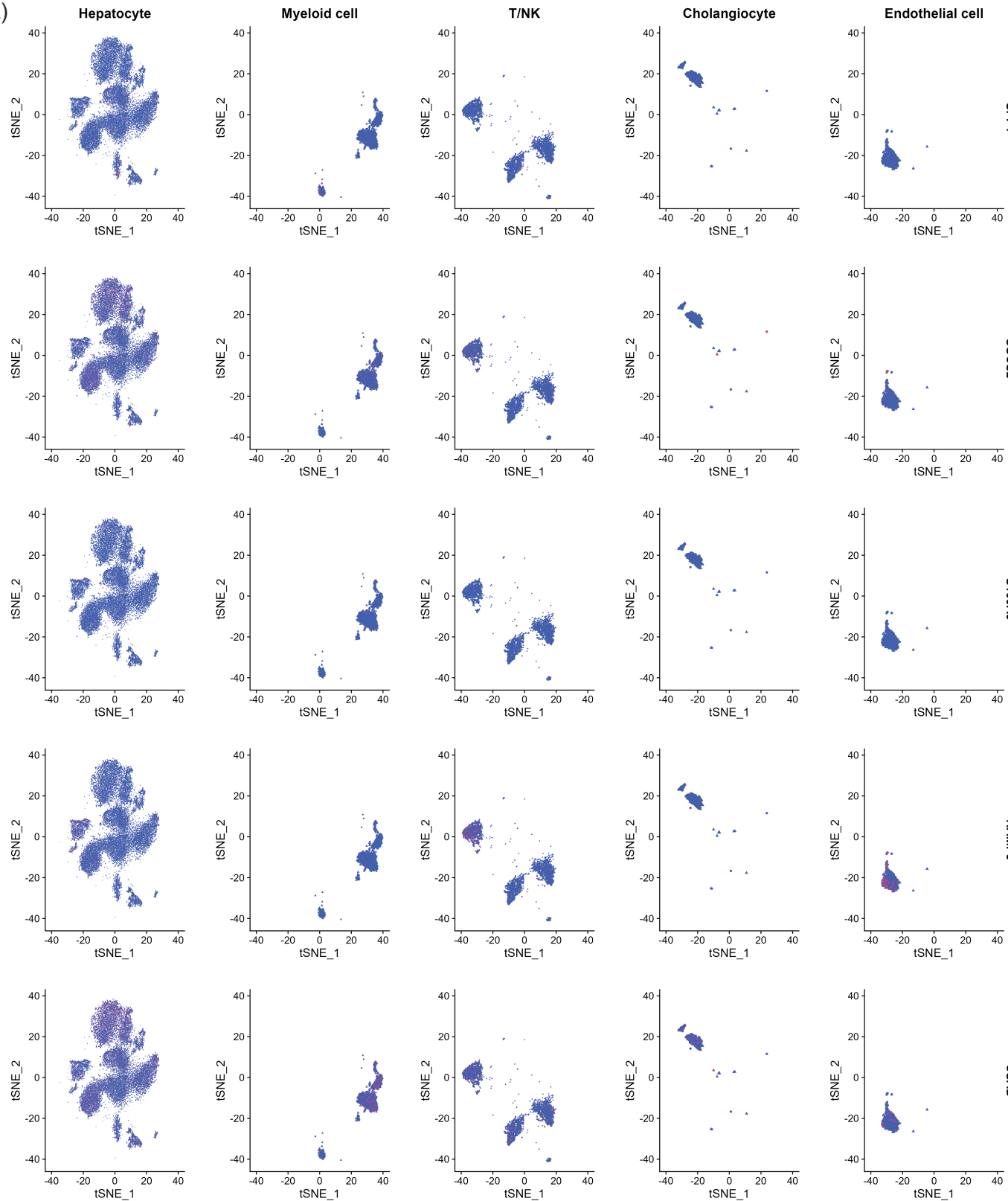

(B)

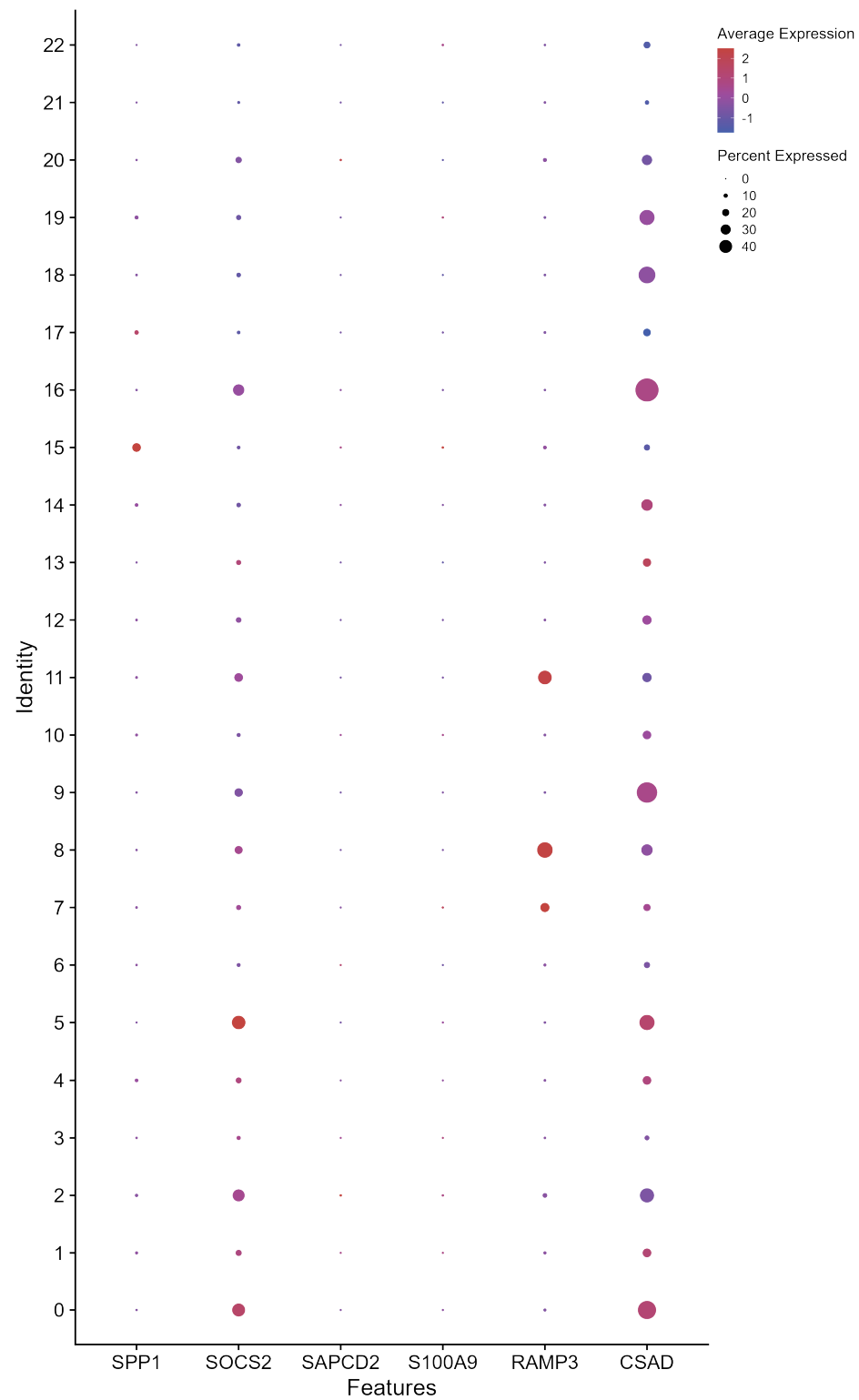

(C)

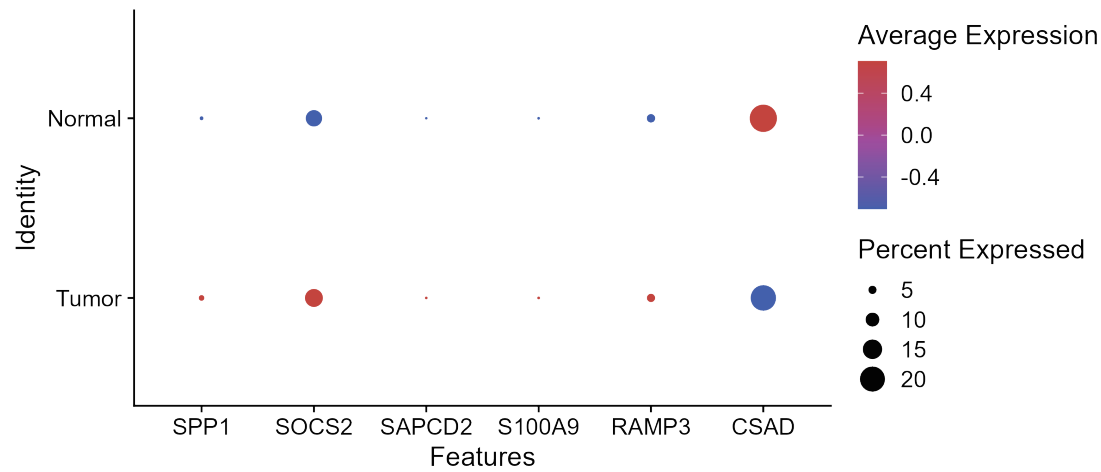

(D)

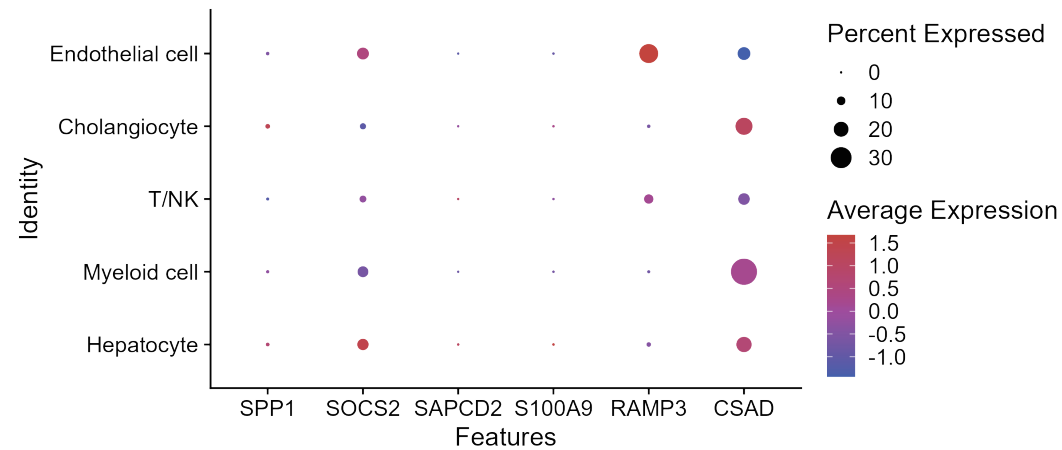

Supplement: Supplemental Information 4 — (A) t-SNE plots show the distribution in different cell subpopulations. Dot plots show the expression of selected genes (SPP1, SOCS2, SAPCD2, S100A9, RAMP3, and CSAD) in different clusters(B), in normal and tumor tissues(C), and in different cell subpopulations(D). [file peerj-12-17002-s004.pdf]

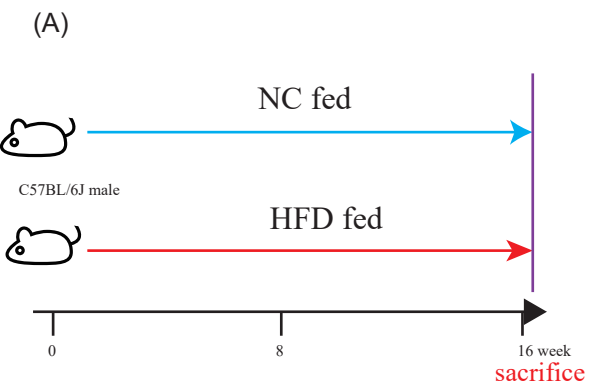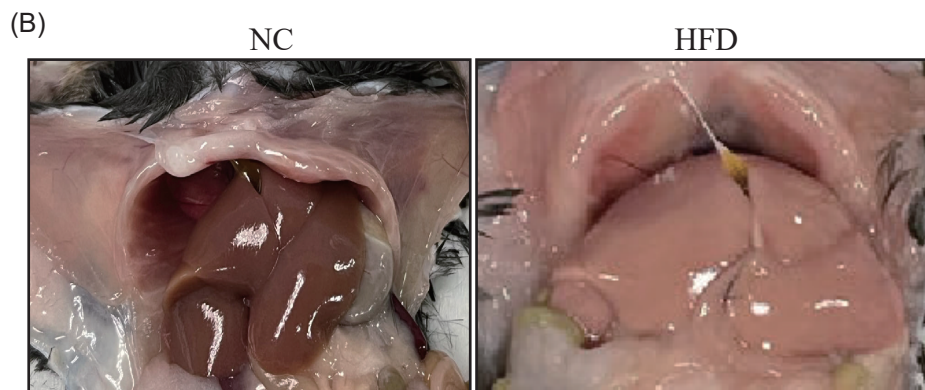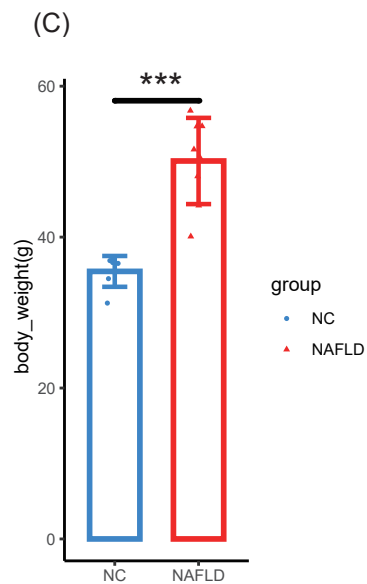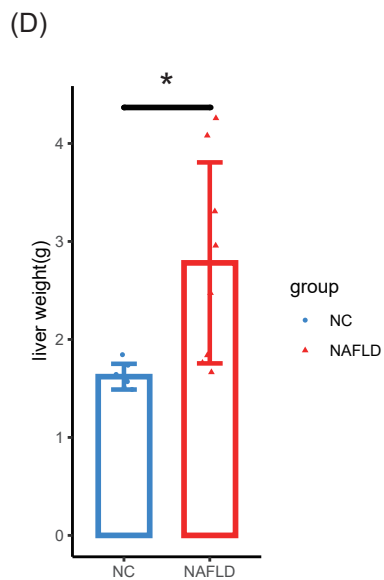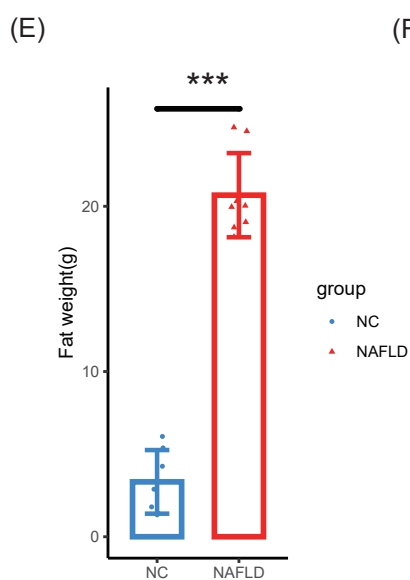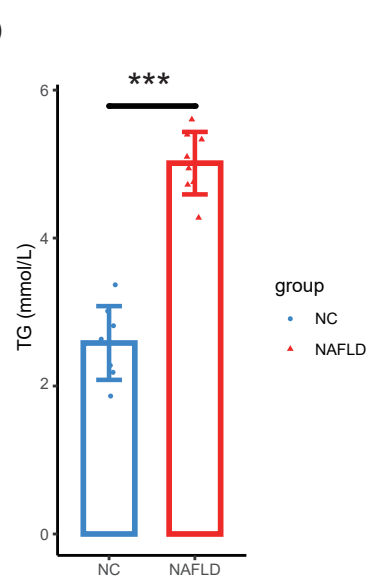

Supplement: Supplemental Information 5 — (A) Flow chart of high-fat diet-induced NAFLD in mice. Liver appearance (B), body weight (C), liver weight (D), fat weight (E), and serum triglycerides (F) at 16 weeks in high-fat and normal diet-fed mice. [file peerj-12-17002-s005.pdf]

(A)

**Scale independence**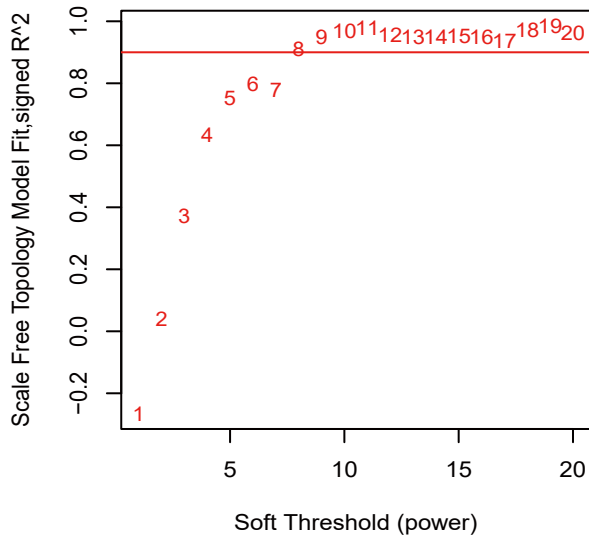

(B)

**Mean connectivity**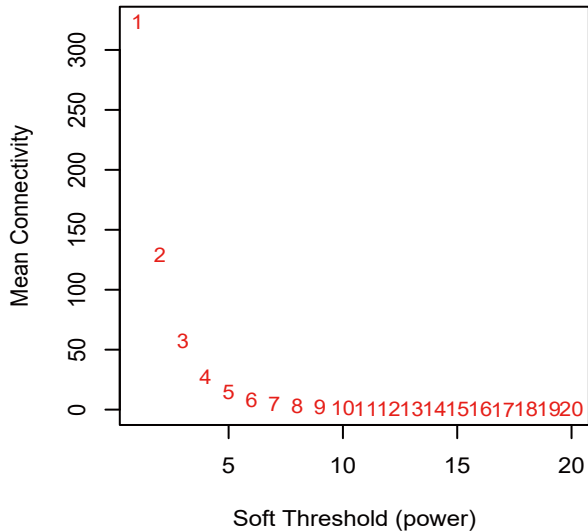

Supplement: Supplemental Information 6 — Utilizing WGCNA, gene co-expression networks for HCC patients were constructed. The prevalence and tendencies of scale-free topological model fit(A) and average connectivity (B). [file peerj-12-17002-s006.pdf]
