# Supplementary material for: Integrating single-cell and bulk sequencing data to identify glycosylation-based genes in non-alcoholic fatty liver disease-associated hepatocellular carcinoma
Source: PeerJ. 2024 Mar 18;12:e17002. doi: 10.7717/peerj.17002 (PMC10956522; doi:10.7717/peerj.17002)
Supplement: Supplemental Information 7 — The bar plot displays the distribution and differences in clinical information between cluster 1 and cluster 2, including cancer type, Child-Pugh grade, gender, histological grade, medical history, TNM staging, overall stage, and vascular invasion. [file peerj-12-17002-s007.pdf]

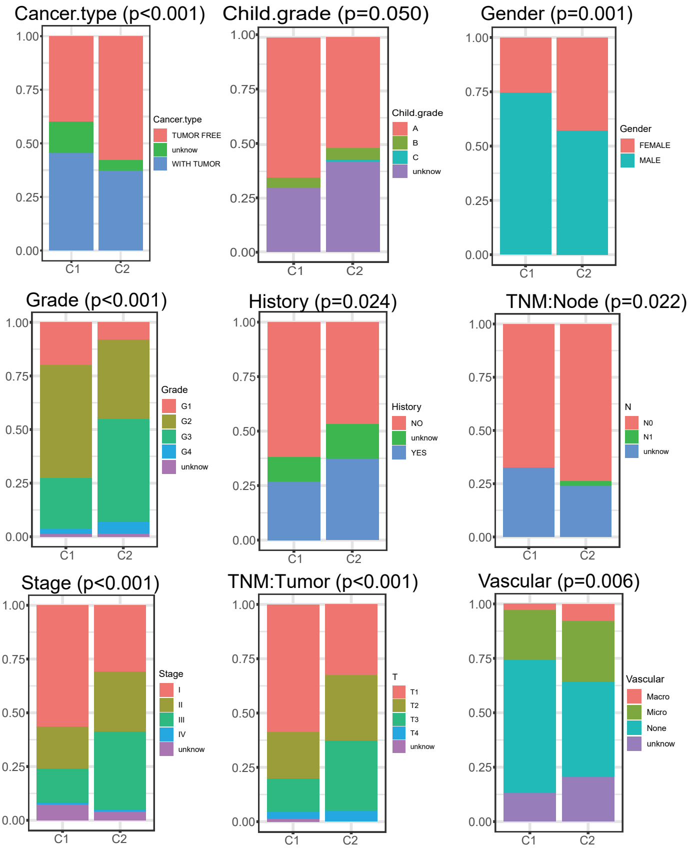

Cancer.type (p<0.001)
Cancer.type
TUMOR FREE
unknow
WITH TUMOR
Child.grade (p=0.050)
Child.grade
A
B
C
unknow
Gender (p=0.001)
Gender
FEMALE
MALE
Grade (p<0.001)
Grade
G1
G2
G3
G4
unknow
History (p=0.024)
History
NO
unknow
YES
TNM:Node (p=0.022)
N
N0
N1
unknow
Stage (p<0.001)
Stage
I
II
III
IV
unknow
TNM:Tumor (p<0.001)
T
T1
T2
T3
T4
unknow
Vascular (p=0.006)
Vascular
Macro
Micro
None
unknow
0.00
0.25
0.50
0.75
1.00
C1
C2
