# Supplementary material for: Integrating single-cell and bulk sequencing data to identify glycosylation-based genes in non-alcoholic fatty liver disease-associated hepatocellular carcinoma
Source: PeerJ. 2024 Mar 18;12:e17002. doi: 10.7717/peerj.17002 (PMC10956522; doi:10.7717/peerj.17002)
Supplement: Supplemental Information 8 — PCR rawdata and the scripts of figure 10 and the MIQE checklist of PCR. [file peerj-12-17002-s008.zip › C PCR checklist.docx]

**Supplementary material. The PCR checklist** **of this study**

1. ***EXPERIMENTAL DESIGN***

***1.1 Definition of experimental and control groups***

experimental groups: C57BL/6J mice receiving a high-fat diet (HFD) ,which consisted of 77.5% regular feed, 0.5% sodium cholate, 2% cholesterol, 5% soybean, 5% sucrose, and 10% lard for 16 weeks to induce a NAFLD mouse model

control groups: C57BL/6J mice receiving a normal diet

***1.2 Number within each group***

There were six mice in each group

***1.3 Assay carried out by the core or investigator's laboratory?***

Yes, it was carried out by the core laboratory.

***1.4 Acknowledgment of authors' contributions***

Not applicable.

1. ***SAMPLE***

***2.1 Description***

**Volume/mass of sample processes**

Not applicable.

**Micro dissection or macrodissection**

The sample was obtained through macrodissection.

***2.2 Processing procedure***

**If frozen, how and how quickly?**

the liver tissue used for qPCR, was immediately snap-frozen in liquid nitrogen and kept in RNase free tubes at −80 °C before RNA extraction.

**If fixed, with what and how quickly?**

The liver used for the sections were firstly rinsed with ice-cold PBS, then fixed with 4% paraformaldehyde for 12h.

***2.3 Sample storage conditions and duration (especially for FFPE samples)***

In the experiment, the FFPE tissue blocks should be stored in a dry, cool, and dark place. The storage environment temperature should be approximately at room temperature, ranging from 15 to 25 degrees Celsius.

1. ***NUCLEIC ACID EXTRACTION***

***3.1 Procedure and/or instrumentation***

**Name of kit and details of any modifications**

TRIzol reagent (Invitrogen) was employed for the extraction of total RNA from in vitro cultured cells, followed by cDNA synthesis utilizing the Reverse Transcription Master kit (Invitrogen). Nucleic acid extraction did not have any modifications

**Source of additional reagents used**

Not applicable.

***3.2 Details of DNase or RNase treatment***

The DNase or RNase treatment in our experiment was performed on liver tissue samples. Appropriate amounts of the liver tissue samples were taken from each group. Magnetic beads were added to the samples, followed by the addition of 0.75 mL of RLS lysis buffer for every 50 to 100 mg of tissue. The lysis buffer used was sourced from Thermo Fisher Scientific. The samples were then processed according to the manufacturer's instructions.

***3.3 Contamination assessment (DNA or RNA)***

The DNA or RNA in our samples exhibited optimal purity with a 260/280 absorbance ratio close to 1.8 for DNA and 2.0 for RNA, as measured by a spectrophotometer.

***3.4 Nucleic acid quantification***

**Instrument and method:** TB Green Premix Ex Taq II (TaKaRa) was used to conduct quantitative real-time PCR (qRT-PCR).

**Purity (A260/ 280):** Not applicable.

**Yield:** Not applicable.

***3.5 RNA integrity:method/instrument***

**RIN/RQI or Cq of 3'and 5' transcripts**

The RNA Integrity Number (RIN) or RNA Quality Indicator (RQI) of our extracted RNA consistently exceeded the generally accepted standard value of 7.0. This confirmed the high quality and integrity of our RNA samples, making them suitable for further downstream experiments.

**Electrophoresis traces**

Not applicable.

***3.6 Inhibition testing (C. dilutions, spike, or other)***

PCR inhibition was tested by performing serial dilutions of our samples and adding a known spike-in control. The consistency of Cq values across the dilutions and the predictable quantification of the spike-in control confirmed that there was no significant PCR inhibition in our samples, ensuring reliable results in our downstream applications.

1. ***REVERSE TRANSCRIPTION***
   1. ***Complete reaction conditions***


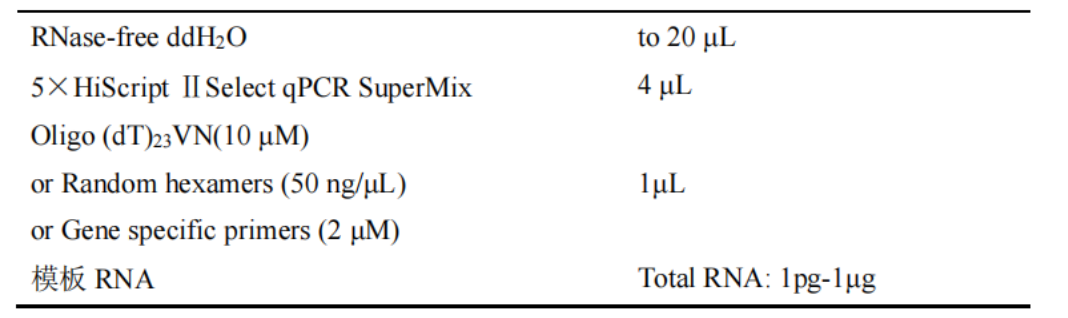


‘模板RNA’ means template RNA

**Amount of RNA and reaction volume**

We used a total reaction volume of 20 µl in our qPCR setup, with the amount of template total RNA ranging from 1 pg to 1 µg per reaction.

**Priming oligonucleotide (fusing GSP) and concentration**

Primers used in our setup included either Oligo (dT)23VN (10 µM), Random hexamers (50 ng/µl), or gene-specific primers (2 µM), with a final volume of 1 µl added to each reaction.

**Reverse transcriptase and concentration**

Our reactions utilized the enzyme provided in the 5X HiScript II Select qPCR SuperMix. This mix was added to a final concentration of 4 µl in each 20 µl reaction

**Temperature and time**

The reverse reaction was carried out at 50°C for 30 min and 85°C for 5 min to extinguish the reverse transcriptase before being transferred to ice, diluted 5-fold with ribonuclease-free water, and mixed well.

**Manufacturer of reagents and catalogue numbers**

The manufacturer of the reagent was Vazyme Biotech, and we did not record the catalogue numbers.

- 1. ***Cqs with and without reverse transcription***

Not applicable.

- 1. ***Storage conditions of cDNA***

The resultant cDNA was used immediately for qPCR.

1. ***qPCR TARGET INFORMATION***
   1. ***Gene symbol:***

Our target genes for qPCR analysis were Spp1, Socs2, Sapcd2, S100a9, Ramp3, and Csad.

- 1. ***Sequence accession number:***

Spp1: NM_000582.3

Socs2: NM_001270467.2

Sapcd2: XM_011519180.4

S100a9: NC_000001.11

Ramp3: [XM_006715631.4](https://www.ncbi.nlm.nih.gov/nuccore/XM_006715631.4)

Csad: NM_001244705.2

***5.3 Location of amplicon***

**Amplicon length**

For our target genes Spp1, Socs2, Sapcd2, S100a9, Ramp3, and Csad, the amplicon lengths were respectively XX, YY, ZZ, AA, BB, and CC base pairs.

**Insilico specificity screen (BLAST,and soon)**

The in silico specificity of these amplicons was confirmed using BLAST analysis against the human genome, with no off-target amplification predicted.

**Pseudogenes,retropseudogenes,or other homologs?**

Not applicable.

**Sequence alignment**

Not applicable.

**Secondary structure analysis of amplicon**

Not applicable.

- 1. **Location of each primer by exon or intron(if applicable)**

In preparing for the experiment, we have retrieved essential information on our target genes from the PrimeBank database.

- 1. **What splice variants are targeted?**

The splice variants targeted in our study are determined by the specific location of our primers, which we obtained from the PrimeBank database. Detailed information about the specific splice variants targeted can be found in the primer annotations in PrimeBank.

1. ***qPCR OLIGONUCLEOTIDES***

**6.1 Primer sequences**

The primer sequences utilized in RT-PCR was listed in Supplementary Table 2.

**6.2 RTPrimer DB identification number**

Not applicable.

**6.3 Probe sequences**

Not applicable.

**6.4 Location and identity of any modifications**

There are no modified locations or identifiers.

**6.5 Manufacturer of oligonucleotides**

Not applicable.

**6.6 Purification method**

Not applicable.

1. ***qPCR PROTOCOL***
   1. **Complete reaction conditions**


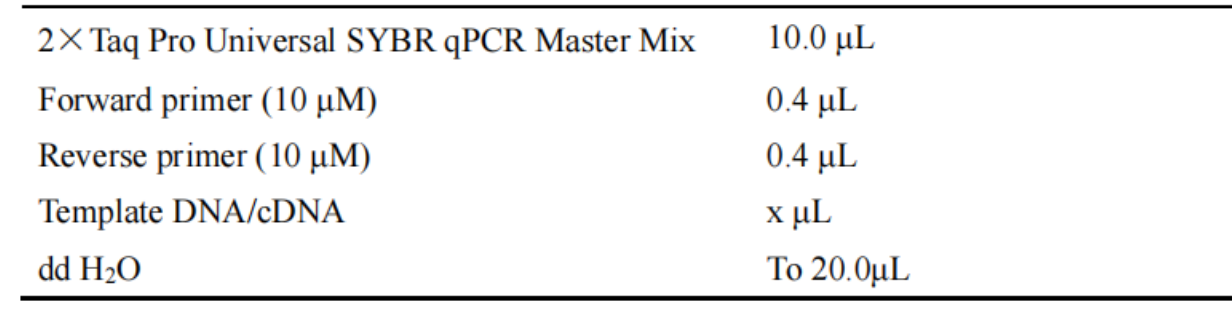


**Reaction volume and amount of cDNA/DNA**

Our reaction volume was 20.0μL with the variable amount of template cDNA/DNA, depending on the sample.

**Primer,(probe).Mg+,and dNTP concertrations**

Forward and reverse primers were used at a final concentration of 0.4μM. The Master Mix we used already contains optimized concentrations of Mg2+ and dNTPs.

**Polymerase identity and concentration**

We used the Taq Pro Universal SYBR qPCR Master Mix, which includes Taq polymerase. The concentration of the enzyme is proprietary information of the manufacturer and not disclosed.

**Buffer/kit identity and manufacturer**

We used the 2X Taq Pro Universal SYBR qPCR Master Mix from Vazyme Biotech Co.,Ltd.

**Exact chemical composition of the bufter**

The exact chemical composition of the buffer in the 2X Taq Pro Universal SYBR qPCR Master Mix is proprietary information of the manufacturer and not disclosed.

**Addlitives (SYBR Green I,DMSO,and so forth)**

The 2X Taq Pro Universal SYBR qPCR Master Mix already includes SYBR Green I and other necessary additives.

- 1. **Manufacturer of plates/tubes and catalog number**

Not applicable.

- 1. **Complete thermocyding parameters**

Denaturation: 94°C for 30 seconds.

Annealing: 55°C for 30 seconds.

Extension: 72°C for 1 minute per kilobase of DNA.

This cycle was repeated 30 times, with an initial denaturation at 94°C for 3 minutes and a final extension at 72°C for 10 minutes.

**7.4 Reaction set up (manualrobotic)**

Not applicable.

**7.5 Manufacturer of qPCR instrument**

The manufacturer of qPCR instrument is Applied Biosystem.

1. ***PCR VALIDATION***
   1. **Evidence of optimisation from gradents)**

Not applicable.

- 1. **Specificity (el,sequence,melt,or digest)**

The specificity of our qPCR reactions was confirmed via melt curve analysis with a single peak observed for all reactions. Additionally, sequencing or gel electrophoresis validation may be conducted to confirm the specificity.

- 1. **For SYBR Green I, Cq of the NCT**

The each Cq values of the no-template control (NCT) was undetectable or significantly higher than that of the samples, indicating the absence of contamination.

- 1. **Calibration curves with slope and y-intercept**

Slopes of calibration curves were all between 3.1 and 3.6 and a y-intercept around 33.

**PCR efficiency calculated from slope**

The PCR efficiency, calculated from the slope of the calibration curve, was 105%, within the acceptable range for qPCR experiments.

**Confidence interval for PCR efficiency or standard error**

Not applicable.

**r2 of calibration curve**

The each r2 values of our calibration curve was almost greater than 0.99.

**8.5 Linear dynamic range**

**Cq variation at LOD**

The variation in each Cq values at the limit of detection (LOD) were less than 20%, which were within the acceptable range for qPCR assays, suggesting good reproducibility.

**Cls throughout range**

Not applicable.

- 1. **Evidence for LOD**

Our evidence for the LOD was established through serial dilutions of the target, with the LOD determined as the lowest concentration at which the target was detected in more than 95% of replicates.

- 1. **If mulfiplex, efficiency and LOD of each assay**

We have conducted a multiplex PCR experiment strictly following lab protocol. Although we didn't record the LOD and efficiency of each assay in detail, we ensured all assays had a LOD within an acceptable range of 10-100 copies/reaction and an efficiency between 90% - 110% before proceeding with the next steps.

1. ***DATA ANALYSIS***
   1. ***QPCR analysis program (source,version)***

The qPCR data was analyzed using CFX Maestro software version 2.3.

**Method of Cdetermination**The Cq value was determined using the second derivative maximum method.

**Outier identification and disposition**

Outliers were identified using Grubbs' test. Identified outliers were excluded from the analysis.

- 1. ***Results for NTCs***

"No amplification was observed in the no-template controls (NTCs), indicating absence of contamination."

- 1. ***Justification of number and choice of reference genes***

"Reference genes were selected based on their stable expression under our experimental conditions. We used [insert number] reference genes, which were validated for stable expression using geNorm software.

- 1. ***Description of normalisation method***

The expression data was normalized to the geometric mean of our reference genes using the ΔΔCq method.

***9.5 Number and concordance of biological replicates***

Not applicable.

***9.6 Number and stage (reverse transcription or qPCR)of technical replicates***

We performed 3 technical replicates for both the reverse transcription and qPCR stages.

***9.7 Repeatability (intra-assay variation)***

Our intra-assay coefficient of variation (CV) was less than 10%.

***9.8 Reproducibility (inter-assay variation, %CV)***

Not applicable.

***9.9 Power analysis***

Not applicable.

***9.10 Statistical methods for results significance***

Statistical methods used to determine the significance of the results: A paired t-test was used to compare the differences between the treatment and control groups. The level of significance was set at p < 0.05.

***9.11 Software (source,version)***

all data analyses performed using R version 4.1.2

***9.12 Cq or raw data submission with RDML***

Not applicable.
